# Response to Malaria Epidemics in Africa

**DOI:** 10.3201/eid1305.061333

**Published:** 2007-05

**Authors:** Tarekegn A. Abeku

**Affiliations:** * London School of Hygiene & Tropical Medicine, London, UK; † Erasmus University Medical Center Rotterdam, Rotterdam, the Netherlands

**Keywords:** Malaria, epidemics, sentinel surveillance, prevention and control, geographic information systems, perspective

## Abstract

Malaria epidemics affect nonimmune populations in many highland and semi-arid areas of Africa. Effective prevention of these epidemics is challenging, particularly in the highlands, where predictive accuracy of indicators is not sufficiently high to allow decisions involving expensive measures such as indoor residual spraying of insecticides. Advances in geographic information systems have proved useful in stratification of areas to guide selective targeting of interventions, including barrier application of insecticides in transmission foci to prevent spread of infection. Because rainfall is associated with epidemics in semi-arid areas, early warning methods based on seasonal climate predictions have been proposed. For most areas, response measures should focus on early recognition of anomalies and rapid mass drug administration. Vector control measures are useful if abnormal transmission is highly likely and if they can be selectively implemented at the early stages of an outbreak.

## Abstract 2

Actions should focus on early recognition of abnormal transmission and rapid deployment of mass drug administration.

Malaria epidemics frequently affect highlands and semi-arid areas where populations lack immunity. Rapid response to these epidemics can be made where effective surveillance systems are in place for early recognition of disease incidence anomalies. Recent advances in research on malaria early warning systems are potentially useful to reduce the effects of epidemics, but they are associated with challenges that face most developing countries. Here, different intervention decisions will be discussed within the context of research findings relevant to epidemic malaria and implementation capacities of epidemic-prone countries.

## Highlands and Semi-arid Areas

Most malaria epidemics follow abnormal weather conditions, often in combination with other causes, including increased resistance of the parasite to antimalarial drugs, population movement due to seasonal labor and civil unrest, and reduced malaria control operations, in particular, the cessation of regular vector control (*1*).

In highlands, transmission is unstable due to fluctuations in temperatures that are normally low (*2*). Temperature affects duration of the sporogonic cycle of the *Plasmodium* parasite within the *Anopheles* vector, survival and feeding frequency of the adult female, and duration of the aquatic stages. Most of the epidemics affecting highlands that support short, annual, transmission are superimposed over normal seasonal increases in malaria incidence, a phenomenon that makes early detection difficult. Other areas experience occasional transmission in specific years with more pronounced levels of illness and death, and substantial spatial and temporal variations (*3*). In addition to explosive epidemics, highland areas in Africa have shown a trend of increasing malaria transmission in recent years (*4*). This trend has important implications for choosing response mechanisms.

Semi-arid areas, on the other hand, have mostly warm climates, and epidemics are associated with anomalous rainfall, which causes increases in vector breeding and survival. In Botswana, more than two thirds of the variability observed between years in malaria incidence during January–May could be explained by variation in rainfall during December–February (*5*). A major epidemic that affected semi-arid regions of northeastern Kenya in January–May 1998 was reportedly caused by abnormal rainfall and floods during November–December 1997 (*6*). In these areas, monitoring rainfall can provide a fairly accurate forecast of transmission risk (*5**,**7**,**8*).

## Preventive Interventions

The type and targets of interventions depend on the forecast probability, available resources, and the timing of the events or available lead time. In most cases, highly accurate forecasting is not possible as yet (e.g., in highlands); hence, emphasis should be placed on improving surveillance for early detection of abnormal incidence to minimize delays in responding (*9*).

Malaria control programs are usually faced with uncertainties regarding decisions on whether seasonal preventive measures should be used routinely in areas known to be at risk due to their geographic characteristics or whether developing mechanisms for rapid response is a better strategy. Although decisions have to be made rapidly in epidemic situations, they also have to be economically justifiable. Interventions that are appropriate in semi-arid areas can be difficult to implement or may be less useful in highland situations. In particular, measures aiming at prevention are more suited to semi-arid areas and sometimes to highlands with moderate malaria endemicity, rather than to highlands that are normally malaria free and only occasionally affected by epidemics.

In most highlands with substantial interannual variability of incidence, measures such as regular indoor residual spraying (IRS) of insecticides for preventive purposes may be an unnecessary waste of resources. However, IRS on an annual basis may be justified in some highland fringe areas. For example, in Madagascar, annual IRS was restored during 1993–1998 to reverse the spread of epidemic malaria that reappeared following recolonization of the central highlands by *A .funestus*, which had disappeared after effective control campaigns (*10**,**11*). After 5 years of spraying DDT mostly in areas between 1,000 m and 1,500 m in altitude, vector density and malaria prevalence rates were greatly reduced. Annual IRS campaigns in the 1950s in the epidemic-affected highlands of Kenya have also produced similar results (*12**,**13*). Selective application of insecticides for a limited number of years in areas most at risk for unstable transmission, followed by focal use as and when required, should be considered, especially in areas bordering malaria-endemic lowlands or in transmission foci in valleys in highland regions.

Highland valleys are often the source of malaria infection. A survey in an altitudinal transect in the Usambara mountains in Tanzania has shown the importance of local topography in explaining variations in splenomegaly among residents (*14*). Not surprisingly, altitude correctly predicted whether an occupant had an enlarged spleen in 73% of households. Moreover, when land where water was likely to accumulate within 400 m of each household was included in the model, prediction significantly improved in areas with an altitude from 1,000 to 1,200 m, where malaria transmission is unstable. In the western Kenyan highlands, the indoor density of *A. gambiae* s.s. vectors has been shown to be negatively associated with distance from swamps (*15*). Areas near man-made breeding sources also have high transmission risks. For example, human activities such as brick making have played a role in creating vector-breeding sites during the dry seasons (*16*). A spatial analysis of the distribution of *P. falciparum* in the highlands of Kenya has indicated that prevalence of infection and parasite densities decreased with distance from valley bottoms (*17*). These foci maintain low levels of transmission through the dry periods and are a potential source of infection when weather and other conditions favor spread of the disease to surrounding highlands. Selective annual insecticide spraying of houses in and around these valley bottoms and areas in the vicinity of man-made transmission sources may help protect the populations in the highlands during transmission seasons. An effective insecticide with a long residual life (such as DDT) should be used. The potential use of geographic information systems and remote sensing technologies to map transmission foci and risk factors and to guide targeting of interventions has been extensively reviewed (*18*). Spatial epidemic risk maps, based on the climate profiles of epidemic-affected localities, have been proposed for highland areas in the Horn of Africa and East Africa (*2*). Static spatial maps have been used for general stratification of areas and, in some cases, for regular interventions in countries such as Madagascar (*10*), South Africa (*19*), and India (*20*).

In the past few years, several international collaborative efforts have been initiated to develop and test temporal risk maps based on rainfall anomalies by using remote sensing technologies, with special applicability in semi-arid areas (*7*). Continually updated rainfall anomaly maps are available free of charge from the websites of the Famine Early Warning Systems Africa Data Dissemination Service (http://igskmncnwb015.cr.usgs.gov/adds) and the International Research Institute for Climate and Society (http://iridl.ldeo.columbia.edu/maproom/.Health/.Regional/.Africa/.Malaria). The practical use of these technologies for early warning of abnormal transmission and selective application of prevention or preparedness is yet to be fully evaluated, although encouraging results have been documented in southern Africa, especially in Botswana (*7*). Because rainfall variability in Botswana has high predictability of interannual variations in malaria incidence (*5*), an early warning system has been proposed to provide probabilistic forecasts of anomalously high or low incidence in semi-arid areas on the basis of seasonal precipitation forecasts (*8*). Although claims have been made that the system can add up to 4 months of warning over methods that use observed precipitation, the system’s sustainable applicability for targeting preventive measures remains to be seen.

In the highlands, monitoring temperature anomalies can provide crude forecasts. Studies have shown that major epidemics in the 1980s and early 1990s in Ethiopia were significantly more often preceded by a month of abnormally high minimum temperature than would be expected by random chance (*3*). Similarly, higher than average temperatures in Zimbabwe were associated with severity of epidemics and deaths in the following year (*21*). Studies involving concomitant longitudinal follow-up of weather patterns, indoor resting densities of vectors, and malaria incidence are ongoing in highland sites in Kenya and Uganda to clarify and quantify epidemic-triggering mechanisms (*22*).

Several studies have shown epidemic malaria to be associated with El Niño events in many parts of the world (*23*). For example, analysis of malaria data from Colombia for 1980–1997 appears to indicate that El Niño events intensify the annual seasonal transmission cycle (*24*). Prediction based on El Niño indicators may prove useful for ensuring availability of resources at national levels, in particular, drugs and insecticides, because of the relatively long lead times (*25*).

## Rapid Assessment

Early detection capacity is essential for effective and rapid containment of epidemics. An important aspect of such capacity is the existence of efficient disease surveillance systems. In East Africa, research has shown that computer-assisted, weekly sentinel surveillance can be successfully implemented at district levels (*22*).

After detection of abnormal increases, mostly increased numbers of clinically diagnosed cases, the actual causes of the increases should be rapidly confirmed through laboratory diagnosis before intervention measures are recommended. Confirmation should be followed by assessing the magnitude and geographic extent of the outbreak, prioritizing areas, and deciding on the types of interventions required. These steps, however trivial they might seem, are nevertheless essential and must be taken within the shortest possible time. In most instances, simple rapid assessment methods are sufficient for sound decisions. As an example, in an epidemic that affected Uasin Gishu District in Kenya, school absenteeism was used as an indicator to determine priority areas for mass treatment of fever (*26*).

More advanced techniques that are economical but powerful and rapid can also be implemented, especially if sampling procedures are adopted in advance of an epidemic event. One such technique is lot quality assurance sampling (LQAS). The LQAS method was first used in industrial sampling to identify batches of products (or lots) with unacceptable number of defective items. In public health, this method has been used to select communities with disease prevalence rates beyond acceptable levels (*27*). Its advantage is that relatively smaller sample sizes are required than in other sampling methods. After an acceptable probability of error and the maximum sample size are determined, the population is sampled until a certain number of infections is exceeded; the sampling then stops and the area is classified as having high prevalence. In Madagascar, this procedure was compared with a conventional sampling plan to select areas where prevalence rates among schoolchildren were at least 15%; such areas were candidates for some specific action (*28*). A plan in which 2 persons were found positive among a random sample of 36, denoted as (36,2), classified communities correctly with 100% sensitivity and 94% specificity. After such assessments, the use of IRS for epidemic control should only be considered if continuation of transmission is anticipated over a long period and if rapid implementation of IRS is feasible at the early phase of an epidemic. The main challenge is that few countries have the capacity to rapidly organize and implement IRS as a meaningful epidemic-containment method.

Sufficient evidence that insecticide-treated nets (ITNs) are beneficial for short-term epidemic control is lacking, so their use should be limited to situations in which their availability and rapid implementation is possible, such as in refugee camps (*25*). Nevertheless, widespread use of ITNs in epidemic-prone areas with moderate malaria endemicity will, in the long run, contribute to transmission reduction and should be encouraged. Larval control may have a limited role in some situations, for example, in semi-arid areas, with well-defined mosquito breeding sites after rainy periods, or in man-made sites such as wells and water storage tanks. Larviciding is likely to be effective when bodies of water cannot be eliminated due to essential economic activities such as brick making (*16*).

Occasionally, widespread epidemics affect large geographic areas such as the highlands of Ethiopia, affected in 1988 (*3*). In such situations, carrying out large-scale preventive IRS operations would be difficult, if not impossible, even if early warning systems are in operation. Health services need to focus on more feasible measures such as strengthening preparedness by stocking drugs and diagnostic materials, closely monitoring changes in malaria incidence, educating communities to seek prompt treatment, classifying areas according to their risk levels, and making contingency plans to rapidly deploy mobile treatment teams.

## Mass Drug Administration and Mass Fever Treatment

Mass drug administration (MDA) is the presumptive treatment of an entire affected population with a therapeutic dose of an antimalarial drug, whereas mass fever treatment (MFT) refers to treatment of febrile patients only. These approaches require that sufficient and appropriate antimalarial agents are available and that mobile treatment teams can be deployed in affected areas in the shortest possible time. In all epidemic situations, rapid distribution of effective treatment in affected communities is recommended. This requires local surveillance and logistics capacity for early detection of abnormal incidence and timely mobilization of resources.

In areas where reliable early warning systems are not in place due to technical, logistical, or other reasons, stocking contingency antimalarial drugs in health units across areas at risk before known transmission seasons provides an alternative approach. Epidemics tend to occur during those seasons of known transmission, which mostly follow the rainy period. Areas historically known to be most at risk should be identified to prioritize health units for drug distribution. If available, risk maps may be used for classification of areas. A practical alternative for inter-area comparisons is to use proxy measures such as adult-to-child ratios of patients attending health facilities. This approach has been used in Kenya to study stability of malaria in the highlands (*29*). Malaria patients admitted to the hospital were classified into 2 age groups: <15 years of age (“children”) and >15 years (“adults”). Depending on the age structure of the developing country populations, the adult-to-child ratio of hospital admissions approached unity for an unstable malaria situation in which adults were as likely as children to be at risk for severe malaria.

MDA has been used alone or in combination with IRS to prevent and control malaria in various settings. The primary objective of this measure in the epidemic control context is to reduce the reservoirs of the parasite by reducing infectiousness to vectors, while providing curative and prophylactic benefits to treated persons. To have the desired effects on transmission, antimalarial drugs with schizontocidal and gametocytocidal effects should be used. In the past, primaquine was given in combination with 4-aminoquinolines for its effect on gametocytes. In a malaria-endemic area in Tanganyika (present day mainland Tanzania), the repeated use of amodiaquine and primaquine combination considerably lowered transmission by reducing the sporozoite rates (*30*). Repeated MDA with proguanil has been shown to substantially reduce transmission in the highlands of western Kenya in the late 1940s (*31*). Although many MDA trials did not interrupt transmission, most succeeded in considerably reducing parasite prevalence, and some showed marked reduction in incidence of cases and deaths (*32*).

Alternatively use of MFT as an important rapid measure, rather than using MDA for the entire population, has been proposed for epidemic control (*25*). Attaining high coverage is crucial; epidemiologically relevant questions include the practicality of diagnosing fever cases in emergency situations and whether a large enough proportion of the population can be treated in this way in order to have a considerable impact on transmission. In Ethiopia, the Ministry of Health guidelines recommend rapid sampling of households to determine the proportion of occupants with illness in the previous 7 days; a cut-off value of 50% would then be used to decide whether to use MFT or MDA (*33*).

The introduction of combination therapy with artemisinin derivatives, which have gametocytocidal effects, has recently led to the hypothesis that its use for large-scale MDA might be a potential malaria control measure (*32*). Artemisinin-based combination therapy (ACT) drugs have been shown to moderately reduce transmission by reducing the duration of gametocyte carriage and the proportion of mosquitoes that are infected by carriers (*34*). In The Gambia, children treated with the combination of chloroquine and artesunate were significantly less infectious to mosquitoes than children treated with chloroquine alone (*35*). Treatment with the combination containing artesunate also significantly reduced the prevalence and density of gametocytes, as well as the duration of gametocyte carriage, although the effect was transient as it did not prevent emergence of mature gametocytes at day 28 after treatment (*35*). Another study in an area with highly seasonal but intense transmission in The Gambia showed that MDA with a single dose of artesunate combined with sulfadoxine-pyrimethamine failed to interrupt transmission overall, but incidence in the first 2 months was significantly lower in treated villages than in control villages (*36*). The failure of MDA to interrupt transmission in the longer term was attributed to the high entomologic inoculation rate in the area. Nevertheless, MDA with a full therapeutic dose of ACT can likely play a major role in the control of epidemics and malaria in areas with a short transmission season (*32*).

Treatment of patients with fever, whether at health facilities or as part of epidemic control, presents the challenge of balancing costs in time and other resources with accuracy of clinical diagnosis. This diagnostic method is particularly less accurate in areas of low endemicity than in areas of high endemicity (*37*), although sensitivity and specificity tend to increase during transmission seasons (*38*). As a result, overdiagnosis of malaria in areas of low transmission remains a major problem, especially when expensive ACT drugs are to be used for treating patients with fever. Furthermore, surveillance systems that rely entirely on data generated from health facilities without laboratory confirmation can lead to false epidemic alerts (unpub. data).

The cost-effectiveness of rapid diagnostic tests in epidemic situations in relation to the use of ACT has been compared with presumptive treatment by using a model based on actual cost data (*39*). The threshold prevalence beyond which treatment based on rapid diagnostic tests becomes more expensive than presumptive treatment was shown to be 21% for artesunate-amodiaquine and 55% for artemether-lumefantrine. During epidemics, the percentage of highland populations infected or incubating infection is usually higher than the threshold for artesunate-amodiaquine. A recent study in western Kenyan highlands showed that nearly 44% of the sampled population were infected over a 10-week period during an epidemic, with adults and children similarly affected (*40*). These observations indicate that relatively less expensive ACT drugs such as artesunate-amodiaquine can be cost-effective when used in MFT without laboratory confirmation. For large-scale epidemics when most of the population are either infectious or incubating the infection, MDA with relatively inexpensive ACT distributed once or repeated within 1–2 weeks can substantially reduce transmission.

The choice of treatment sites largely depends on the magnitude of the epidemic. Treatment at existing health facilities should be given special attention, and health services should ensure that essential drugs for the treatment of both uncomplicated and severe malaria are in stock. In many situations, mobile treatment centers will be required to cover remote rural areas. In Kenya, mobile treatment teams could be assembled in 1 week through a provincial health system to control an outbreak in Uasin Gishu District (*26*).

## Conclusion

Many countries still need to improve their technical and logistics capacity to deal with high demands in resources to prevent or contain malaria epidemics. Nevertheless, better targeting of interventions by using recent technologic advances in spatial analysis and risk mapping, optimal use of computing facilities in disease surveillance and efficient use of information, and better preparedness especially in terms of antimalarial drug stocks will potentially provide a feasible means of effective epidemic control in many countries. In semi-arid areas, early warning methods using actual or probabilistic prediction of rainfall may be used for making decisions to implement prevention measures. In these areas, most vector control measures, including those targeting the aquatic stages of vectors, may be feasible and useful. In the highlands, anomalies in weather patterns, especially minimum temperatures, may be used as crude early warning indicators of malaria epidemics, mainly for preparedness purposes. However, the focus in these areas should be on early detection of abnormal incidence for rapid initiation of response. In normally malaria-free highlands, measures attempting to prevent transmission based on crude forecasts may not be cost-effective or feasible in most situations. At present, the use of IRS and other vector control measures in these areas should be limited to special situations where selective and timely application is feasible to contain proven, ongoing transmission. More research is required to increase our understanding of the genesis of epidemic malaria in the highlands and to develop better predictive models. Research should also focus on ways of developing local epidemic management capacities within the health service systems. The use of relatively inexpensive ACT drugs such as artesunate-amodiaquine for MDA or MFT should be a primary strategy for rapidly reducing transmission in all epidemic situations.
